# Effect of asciminib and vitamin K2 on Abelson tyrosine-kinase-inhibitor-resistant chronic myelogenous leukemia cells

**DOI:** 10.1186/s12885-023-11304-4

**Published:** 2023-09-05

**Authors:** Seiichi Okabe, Akihiko Gotoh

**Affiliations:** Department of Hematology, Tokyo Medical University, 6-7-1 Nishi-shinjuku, Shinjuku-ku, Tokyo, 160-0023 Japan

**Keywords:** Chronic myeloid leukemia, Imatinib resistance, Ponatinib, STAMP inhibitor, Vitamin K2

## Abstract

**Background:**

Abelson (ABL) tyrosine kinase inhibitors (TKIs) are effective against chronic myeloid leukemia (CML); however, many patients develop resistance during ABL TKI therapy. Vitamin K2 (VK2) is a crucial fat-soluble vitamin used to activate hepatic coagulation factors and treat osteoporosis. Although VK2 has demonstrated impressive anticancer activity in various cancer cell lines, it is not known whether VK2 enhances the effects of asciminib, which specifically targets the ABL myristoyl pocket (STAMP) inhibitor.

**Method:**

In this work, we investigated whether VK2 contributed to the development of CML cell lines. We also investigated the efficacy of asciminib and VK2 by using K562, ponatinib-resistant K562 (K562 PR), Ba/F3 BCR–ABL, and T315I point mutant Ba/F3 (Ba/F3 T315I) cells.

**Results:**

Based on data from the Gene Expression Omnibus (GEO) database, gamma-glutamyl carboxylase (GGCX) and vitamin K epoxide reductase complex subunit 1 (VKORC1) were elevated in imatinib-resistant patients (GSE130404). UBIA Prenyltransferase Domain Containing 1 (UBIAD1) was decreased, and K562 PR cells were resistant to ponatinib. In contrast, asciminib inhibited CML cells and ponatinib resistance in a dose-dependent manner. CML cells were suppressed by VK2. Caspase 3/7 activity was also elevated, as was cellular cytotoxicity. Asciminib plus VK2 therapy induced a significantly higher level of cytotoxicity than use of each drug alone. Asciminib and VK2 therapy altered the mitochondrial membrane potential.

**Conclusions:**

Asciminib and VK2 are suggested as a novel treatment for ABL-TKI-resistant cells since they increase treatment efficacy. Additionally, this treatment option has intriguing clinical relevance for patients who are resistant to ABL TKIs.

**Supplementary Information:**

The online version contains supplementary material available at 10.1186/s12885-023-11304-4.

## Background

Chronic myeloid leukemia (CML) is a malignant myeloproliferative disorder characterized by the t(9;22) translocation, known as the Philadelphia (Ph) chromosome [1, 2]. CML progresses via three stages: the chronic phase (CP), accelerated phase, and blast phase [3]. Clinically, CML cases are typically identified during the CP. The tyrosine kinase activity of the BCR::ABL1 protein is dysregulated, and primitive hematopoietic cellular homeostasis systems are changed by BCR::ABL1, which also increases leukemia cell proliferation [1, 2].

The resultant BCR::ABL1 fusion protein is a constitutively active tyrosine kinase that stimulates a number of signaling pathways, all of which cooperate to cause malignant transformation [1, 2]. CML was formerly associated with poor prognoses, but the prognoses of CML patients has improved considerably over the past 20 years because of the introduction of tyrosine kinase inhibitors (TKI) specific for ABL1, such as imatinib, which inhibits BCR::ABL1 kinase activity [3]. However, some patients may still become resistant to ABL TKIs while receiving first-line therapy via a variety of mechanisms, such as ABL kinase domain mutations [3].

There are five ABL TKIs (imatinib, nilotinib, dasatinib, bosutinib, and ponatinib) used in CML treatment available in most nations [4]. Asciminib, also known as ABL001, could transform the standard of care in this population through its novel mechanism of action of specifically targeting the ABL myristoyl pocket (STAMP) inhibitor [5]. Additionally, asciminib was approved for treatment during the CP of CML with the T315I mutation and for patients with CML in the CP who had previously received two or more TKIs [6].

Vitamin K is a fat-soluble vitamin present in a variety of foods and is essential for human health [7]. Blood clotting is also related to vitamin K. Vitamins K1 and K2 are the two primary types present in the human diet [8]. Menaquinone, often known as vitamin K2 (VK2), was first isolated from putrefied fishmeal and is a naturally occurring compound produced by a wide range of microorganisms. VK2 has recently been demonstrated to have outstanding antiproliferative effects in a variety of cancer cells [9–12].

VK2 stimulates anticancer activity; thus, we hypothesized that it would increase ABL TKI efficacy in CML cells, particularly those that are resistant to the drug. In this work, we assessed the anti-CML cell activity of VK2. We also investigated whether co-treatment with the ABL TKI, asciminib, and VK2 would increase CML cytotoxicity.

## Methods

### Reagents

The BCR::ABL1 inhibitor, asciminib (ABL-001), and third generation ABL TKI, ponatinib (AP-24,534), were obtained from Selleck Chemicals (Houston, TX, USA). Stock solutions of asciminib and ponatinib were prepared in dimethyl sulfoxide (DMSO). VK2 (menatetrenone) was purchased from Eisai Co., Ltd. (Bunkyo-ku, Tokyo, Japan) and diluted with culture medium.

### CML cell lines

The CML cell line K562 was obtained from the American Type Culture Collection (ATCC, Manassas, VA, USA). T315I-mutant Ba/F3 cells, BCR::ABL (wild-type)-transfected Ba/F3 cells, and ponatinib-resistant K562 cells (K562 PR) were established previously [13, 14]. All cell lines were grown in Roswell Park Memorial Institute 1640 media (RPMI1640) with 10% fetal calf serum (FCS) at 37 °C in a humid environment with 5% CO_2_.

### Data collection and processing

The microarray data of CP-CML patients (GSE130404) who received imatinib as a frontline treatment and failed to achieve an early molecular response (EMR failure: BCR::ABL1 > 10% (IS) at 3 months) were downloaded from the Gene Expression Omnibus (GEO) database (https://www.ncbi.nlm.nih.gov/geo/). A comparison between EMR failure (n = 13) vs. EMR achieved (n = 83) samples was conducted using the limma analysis bioconductor package [15]. The data were analyzed using the GEO2R website, which is an interactive web tool that allows users to compare two or more groups of samples in a GEO series. The data were downloaded in SOFT format, converted into XLS files, and screened using Microsoft Office Excel 2017 (Microsoft Corporation, Redmond, WA, USA). Genes with a log2 (fold-change) ≥ 1.0 and P < 0.05 were identified as differentially expressed genes.

### Cell proliferation assays

Cells were treated with asciminib and/or with VK2 for 72 h, and then cell viability was evaluated with the CellTiter-Glo™ Luminescent Cell Viability Assay Kit (Promega Corporation, Madison, WI, USA) or with Cell Counting Kit-8 (Dojindo Laboratories, Mashikimachi, Kumamoto, Japan) according to the manufacturer’s instructions. After being cultured for 72 h, the samples were analyzed using an EnSpire Multimode Plate Reader (PerkinElmer, Waltham, MA, USA).

### Determination of caspase 3/7 activities

The Caspase Glo 3/7 Assay Kit (Promega Corporation) was used in accordance with the manufacturer’s instructions to test caspase activity in CML cells. The cells were given the recommended doses of asciminib and/or VK2. After 48 h of treatment, equal amounts of caspase 3/7 reagent were added to each well, and sample luminescence was measured using the EnSpire Multimode Plate Reader (PerkinElmer).

### Cytotoxicity assays

The cells were treated with asciminib and/or VK2 at the recommended concentrations. To determine cytotoxicity, the amount of lactate dehydrogenase (LDH) released from injured cells was measured using the Cytotoxicity LDH Assay Kit with Water-Soluble Tetrazolium (WST) salt (Dojindo Laboratories). Following a 72-h course of therapy, the amount of LDH was analyzed using the EnSpire Multimode Plate Reader (PerkinElmer) according to the manufacturer’s protocol.

### Cell cycle analysis

K562 and K562 PR cells were treated with asciminib (10 nM) and VK2 (10 µM). Following a 24-h treatment period, the BD Cycletest Plus DNA Reagent Kit (Becton-Dickinson, Mountain View, CA, USA) was used to examine cell cycle phases following the manufacturer’s instructions. The effect of DNA content distribution was evaluated using a BD FACSVerse™ Flow Cytometer (Becton-Dickinson) and analyzed using BD FACSuite™ software (Becton-Dickinson).

### Colony formation assay

According to the manufacturer’s instructions, colony formation experiments were conducted using MethoCult® Express (Catalog #04437; STEMCELL Technologies, Vancouver, BC, Canada) methylcellulose-based culture medium according to the manufacturer’s instructions. Briefly, 1 × 10^4^ K562 or K562 PR cells were plated on MethoCult™ Express with the indicated concentrations of asciminib and/or VK2. The plates were incubated at 37 °C and 5% CO_2_ for 7 days. Colony counts were calculated, and imaging was conducted with an EVOS™ FL Digital Inverted Fluorescence Microscope (Thermo Fisher Scientific Inc., Waltham, MA, USA). The results were determined three times, and the mean and standard error were displayed.

### ATP assays

Asciminib (10 nM) and VK2 (10 µM) were applied to CML cells. Intracellular adenosine triphosphate (ATP) concentrations were assessed after treatment for 72 h using the Cell ATP Test Reagent Kit Ver. 2 from TOYO B-Net (Tokyo, Japan) in accordance with the manufacturer’s instructions. Luciferase activity was measured using an EnSpire Multimode Plate Reader (PerkinElmer).

### Enzyme-linked immunosorbent assays (ELISAs)

K562 or K562 PR cells were grown in RPMI media with or without the appropriate amounts of asciminib and/or VK2 to examine proteasome activity. After 48 h, the cells were collected and kept at − 80 °C. The 20 S Proteasome Assay Kit was used to measure proteasome activity (Cayman Chemical Company, Ann Arbor, MI, USA). Luminescence was measured on an EnSpire Multimode Plate Reader (PerkinElmer).

### Analysis of mitochondrial membrane potentials (MMPs)

MMPs were assessed using the Mitochondria Staining Kit (Merck KgaA, Darmstadt, Germany) according to the manufacturer’s instructions. The cells were treated with the indicated concentrations of asciminib and/or VK2. After incubation for 72 h, JC-1 monomers and aggregates were analyzed using the EnSpire Multimode Plate Reader (PerkinElmer).

### Immunoblot analyses

The immunoblot analyses were conducted as previously reported [16, 17]. K562 or K562 PR cells were treated for 24 h with the indicated concentrations of asciminib and/or VK2. Equal amounts of protein mixed with 2× Laemmli sample buffer were loaded onto 4–20% mini protein TGX gels after being lysed using radioimmunoprecipitation lysis buffer (Bio-Rad, Hercules, CA, USA). Resolved proteins were transferred to a polyvinylidene difluoride membrane (Millipore, Billerica, MA, USA). Membranes were blocked and incubated with primary antibodies at room temperature for 1 h. The following primary antibodies were used: cleaved caspase 3 and cleaved poly (ADP-ribose) polymerase (PARP) (Cell Signaling Technology, Danvers, MA, USA), gamma H2AX [p Ser139] (Merck KgaA), and β-actin (Santa Cruz Biotechnology, Santa Cruz, CA, USA). Membranes were incubated with the appropriate secondary antibodies (Cell Signaling Technology) and developed using an enhanced chemiluminescence system (Amersham Phamacia Biotech, Little Chalfont, UK).

### Quantitative real-time reverse transcription-polymerase chain reaction analysis (RT-PCR)

Total RNA was extracted from myeloma samples using the RNAqueous®-4PCR Kit (Life Technologies Japan Ltd., Minato-ku, Tokyo, Japan) and reverse-transcribed using the First-Strand cDNA Synthesis Kit (OriGene Technologies, Rockville, MD, USA). RT-PCR was performed using the Roche Light Cyber 2.0 detection system (Roche Diagnostic Gmbh, Minato-ku, Tokyo, Japan). The expressions of human *gamma-glutamyl carboxylase* (*GGCX*), *vitamin K epoxide reductase complex subunit 1* (*VKORC1*), *UBIA prenyltransferase domain containing 1* (*UBIAD1*), and *β-actin* were quantitated using the SYBR Green PCR Kit (Roche) according to the manufacturer’s protocol. Specific PCR primers were obtained from Takara Bio Inc. (Otsu, Shiga, Japan).

### Statistical analysis

Prism 9 (GraphPad, San Diego, CA, USA) and Microsoft Excel applications were used to examine the presented data. A *t* test was used to determine whether differences in drug treatment groups were statistically significant compared with the control group. Statistical significance was defined as *p < 0.05 and **p < 0.01.

## Results

### Efficacy of ponatinib and asciminib against CML cell lines

**Fig. 1:**
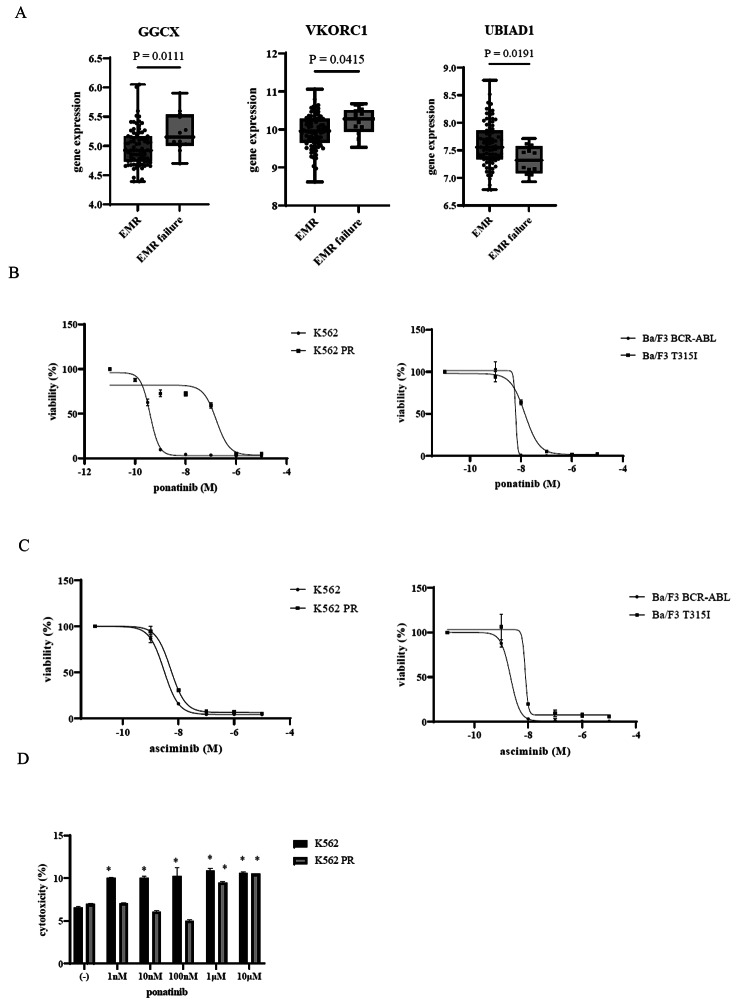
Analysis of expressions of VK2-related genes and the effect of asciminib or ponatinib on ABL TKI-resistant cells. **(A)** Gene expressions of *GGCX*, *VKORC1*, and *UBIAD1*. GEO2R analysis was used to predict gene expression levels. Validation of the *GGCX*, *VKORC1*, and *UBIAD1* gene expressions using the GEO data (GSE130404) was conducted to compare the EMR (*n* = 80) and EMR failure (*n* = 11) groups. The line in the box-and-whisker diagram indicates the median. **(B)** K562, K562 PR, Ba/F3 BCR::ABL, and Ba/F3 T315I cell lines were cultured in RPMI 1640 medium supplemented with 10% FBS with the indicated concentrations of ponatinib for 72 h. Cell growth was evaluated using Cell Counting Kit-8. **(C)** K562, K562 PR, Ba/F3 BCR::ABL, and Ba/F3 T315I cell lines were treated with the indicated concentrations of asciminib for 72 h. Cell growth was evaluated using Cell Counting Kit-8. **(D)** K562 and K562 PR cell lines were treated with ponatinib for 72 h. The cytotoxicity was analyzed in accordance with the manufacturer’s instructions using a Cytotoxicity LDH Assay Kit. ^*^p < 0.05 compared with the control

According to a previous study, individuals in CP CML who do not experience an EMR to imatinib (> 10% BCR::ABL1 after 3 months) will likely have worse outcomes [15]. Hence, using the GEO database, we first investigated the gene expression profiles of patients with EMR failure. *GGCX* and *VKORC1* were elevated in the EMR failure group compared with the EMR achievement group based on the publicly available GSE130404 data. However, *UBIAD1* gene expression was decreased in the EMR failure group (Fig. 1A). A third-generation ABL kinase inhibitor, ponatinib, was created to overcome the gatekeeper T315I mutation [18]. Ponatinib has been approved for the treatment of Ph + ALL patients as well as refractory CML patients [18]. We next investigated the efficacy of different concentrations of ponatinib on ABL TKI-resistant cell lines. Our findings demonstrated that ponatinib inhibited the proliferation of CML cell lines, including Ba/F3 T315I cells, in a dose-dependent manner (IC50: 14.3 nM) (Fig. 1B). In contrast, the ponatinib-resistant cell line, K562 PR, was less sensitive to ponatinib (IC50: 170 nM) (Fig. 1B). Asciminib is a first-in-class ABL kinase inhibitor specifically targeting the STAMP inhibitor [5]. The effectiveness of asciminib against ABL TKI-resistant cells was then examined. Our results showed that asciminib inhibited the proliferation of CML cells, including K562 PR (IC50: 5.2 nM) and Ba/F3 T315I cells (IC50: 7.8 nM), in a dose-dependent manner (Fig. 1C). We then conducted a cytotoxicity analysis because LDH-based tests can be used to measure percentages of dead cells. We found the percentage of dead cells due to cytotoxicity was reduced by ponatinib in K562 PR cells (Fig. 1D).

### Activity of asciminib and VK2 in CML cell lines

**Fig. 2:**
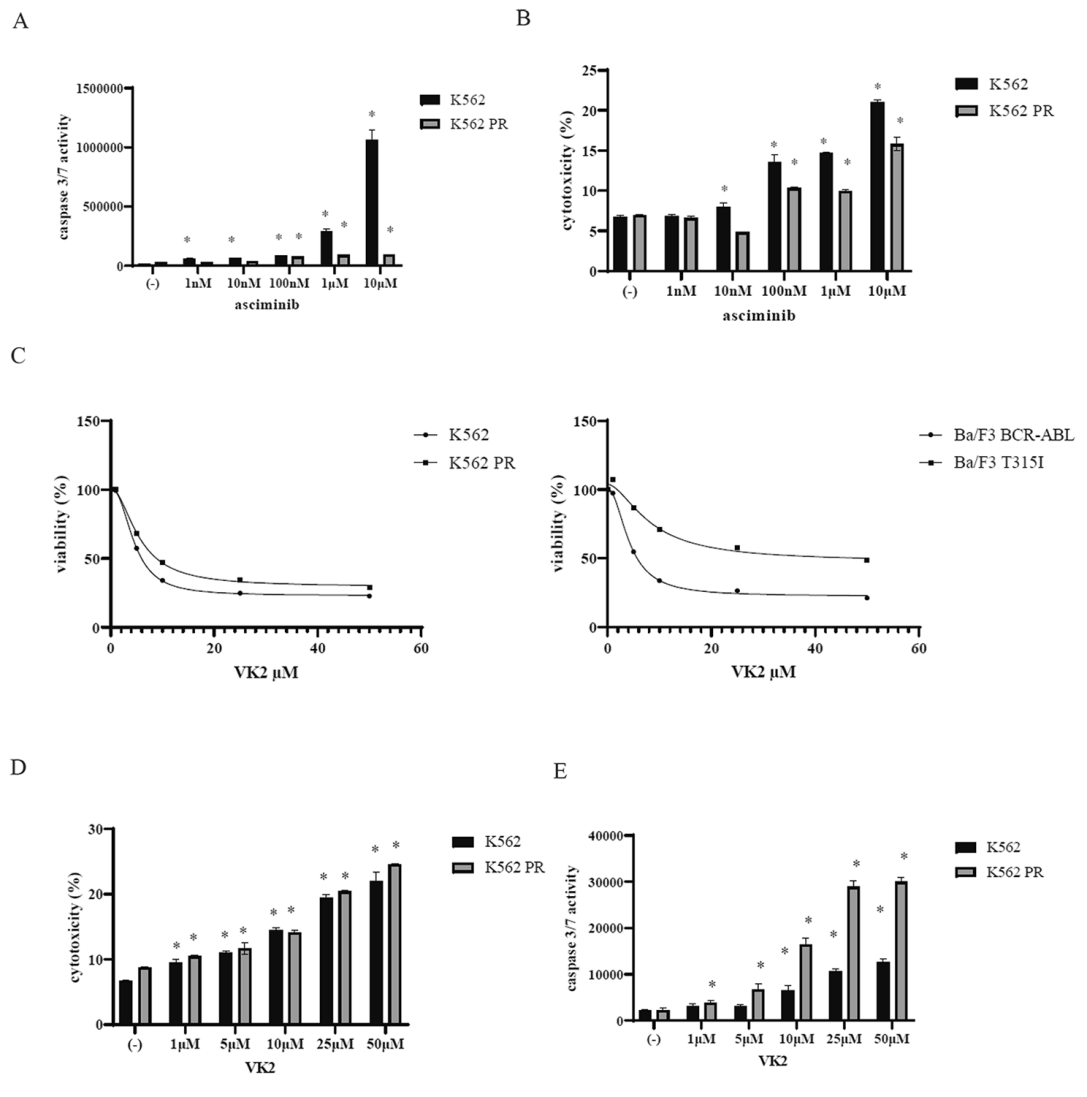
Effects of asciminib or VK2 on the CML cell lines. CML cell lines were incubated with asciminib for 48 or 72 h. Caspase 3/7 activity **(A)** and cytotoxicity **(B)** were evaluated. ^*^p < 0.05 vs. the control. **(C)** CML cell lines were cultured in RPMI 1640 medium supplemented with 10% FBS with the indicated concentrations of VK2 for 72 h. Cell growth was evaluated using the CellTiter-Glo™ Luminescent Cell Viability Assay Kit or Cell Counting Kit-8. **(D)** K562 and K562 PR cell lines were treated with the indicated concentrations of VK2 for 72 h. Cytotoxicity was analyzed using a Cytotoxicity LDH Assay Kit. ^*^p < 0.05 compared with the control. **(E)** K562 and K562 PR cell lines were treated with the indicated concentrations of VK2 for 48 h. Caspase 3/7 activity was analyzed using the Caspase Glo 3/7 Assay Kit. ^*^p < 0.05 compared with the control

Caspase 3/7 activation is a reliable sign of cell death [19]. K562 and K562 PR cells were incubated with the indicated concentrations of asciminib for 48 h. We discovered that asciminib increased caspase 3/7 activity in a dose-dependent manner (Fig. 2A). We next investigated the cytotoxicity of asciminib. K562 and K562 PR cells were incubated with the indicated concentrations of asciminib for 72 h. Asciminib-induced cytotoxicity increased in a dose-dependent manner (Fig. 2B). According to the gene expression profiles, VK2-related genes were involved in ABL TKI resistance, including imatinib resistance. Therefore, we investigated VK2 efficacy by using the ponatinib-resistant K562 cell line and BCR::ABL point-mutant T315I Ba/F3 cells. Our results showed that VK2 inhibited the proliferation of CML cell lines in a dose-dependent manner (IC50: K562, 4.5 µM; K562 PR, 5.5 µM; Ba/F3 BCR–ABL, 4.2 µM; Ba/F3 T315I, 8.3 µM) (Fig. 2C). Cytotoxicity and caspase 3/7 activity were also increased by VK2 treatment in a dose-dependent manner (Fig. 2D and E).

### Analysis of the anticancer effects of asciminib and VK2 by colony formation assays

**Fig. 3:**
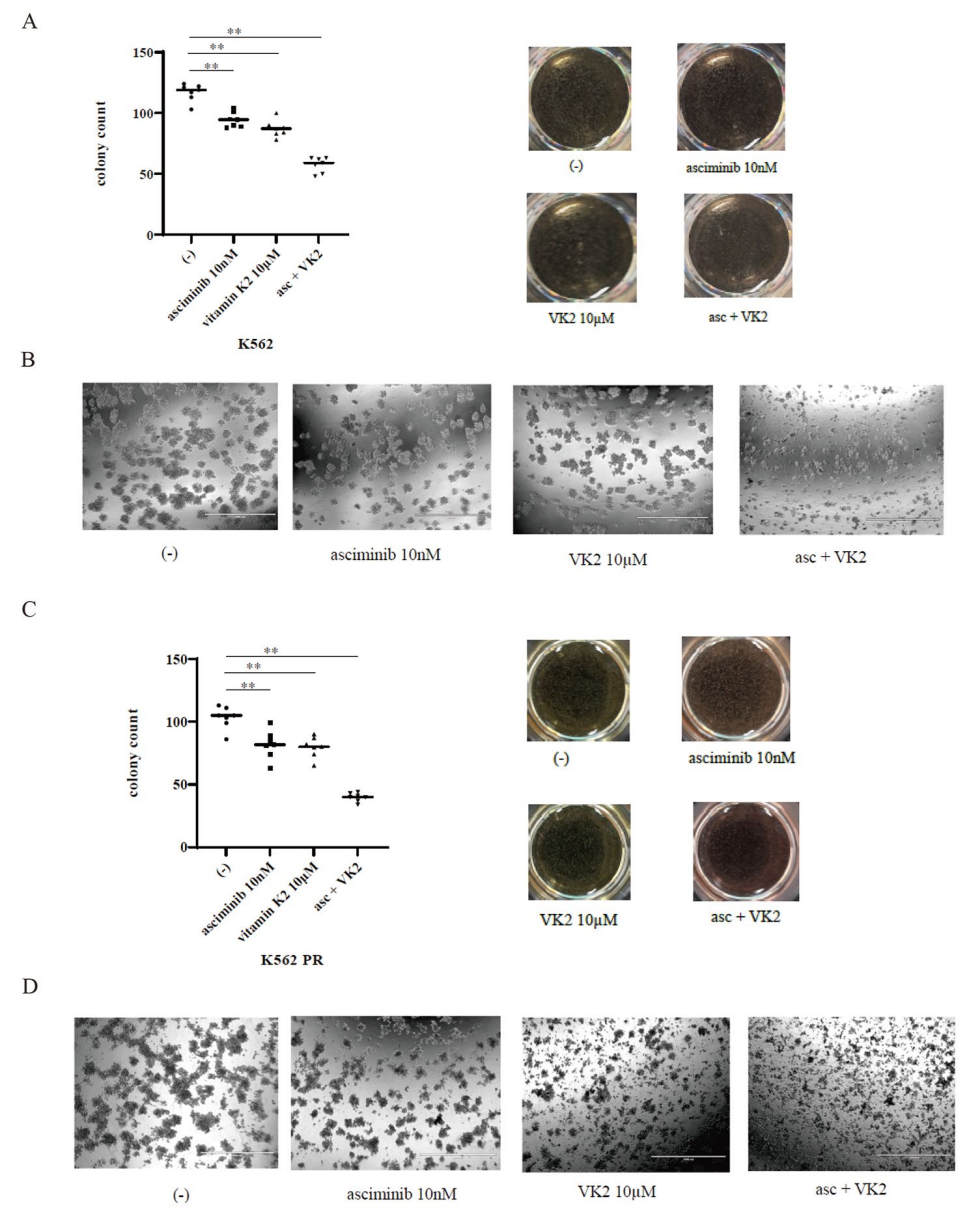
Colony formation assay of the K562 and K562 PR cell lines. K562 **(A, B)** or K562 PR **(C, D)** were treated with 10 nM asciminib and/or 10 µM VK2 for 7 days. The colonies on each dish were photographed using a digital camera and counted using an EVOS™ FL Digital Inverted Fluorescence Microscope. The quantitative graph displaying the colonies formed and representative images are shown. Scale bar: 1,000 μm. ^**^p < 0.01 vs. the asciminib and VK2 treatment groups. The results represent three independent experiments

The colony formation assay is an in vitro quantitative technique used to assess cell survival based on a single cell’s capacity to develop into a colony [20]. To determine the anticancer effects of asciminib and VK2, we investigated the long-term effects of asciminib (10 nM) and VK2 (10 µM) singly and in combination in colony formation assays. We found colony count was reduced by combination of asciminib and VK2 (Fig. 3A). Asciminib and VK2 co-treatment reduced colonies more than either drug used separately, as shown in the bright field image (Fig. 3B). Next, we used K562 PR cells to conduct the colony formation experiment. In Fig. 3C and D, co-treatment with asciminib and VK2 also reduced the clonogenic survival of the CML cell line K562 PR. These results indicated that asciminib and VK2 reduce cell proliferation in CML cell lines, including those that are ponatinib-resistant.

### Efficacy of the asciminib and VK2 combination against CML cells

**Fig. 4:**
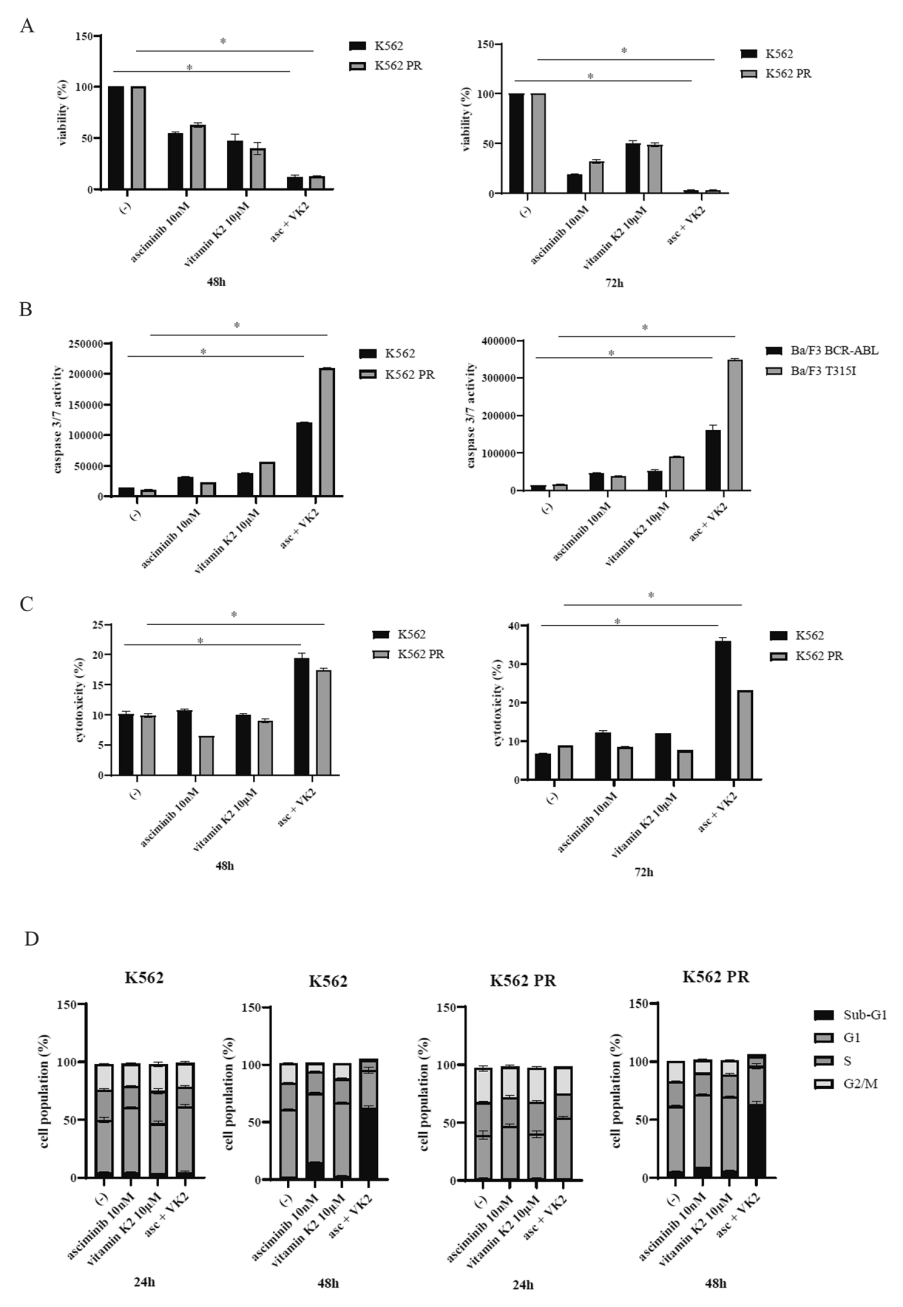
Co-treatment with asciminib and VK2 induced cytotoxicity in the CML cells. CML cell lines (K562 and K562 PR) were incubated with 10 nM asciminib and/or 10 µM VK2 for 48 or 72 h. Cell growth **(A)**, caspase 3/7 activity **(B)**, and cytotoxicity **(C)** were evaluated. ^*^p < 0.05 vs. the control. **(D)** Cell cycle phase profiling was determined using the BD Cycletest™ Plus DNA Reagent Kit using K562 or K562 PR cells treated with 10 nM asciminib and/or 10 µM VK2 for 24 or 48 h. A representative histogram for each condition is shown

Next, we conducted cell proliferation assays to assess the effectiveness of asciminib and VK2 against ABL TKI-resistant or T315I mutant cells. Compared with each drug alone, co-treatment with asciminib and VK2 inhibited cell proliferation, including in ponatinib-resistant K562 PR and Ba/F3 T315I cells, in a time-dependent manner (Fig. 4A, Supplemental Fig. 1A). Co-treatment with lower dosages of asciminib and VK2 slightly reduced the proliferation of K562 PR cells; however, this effect was not observed in the K562 parental cell line (Supplemental Fig. 1B). We also found co-treatment with ponatinib and VK2 inhibited cell proliferation in K562 and K562 PR cells. (Supplemental Fig. 1C). Caspase 3/7 activity and cytotoxicity were increased, including in ponatinib-resistant K562 PR or Ba/F3 T315I cells, compared with each drug alone in a time-dependent manner (Fig. 4B C, Supplemental Fig. 1D). To explore whether asciminib and VK2 mediated the inhibition of CML cell growth via cell cycle signaling, we conducted cell cycle analyses. Treatment with asciminib and VK2 for 24 h statistically increased the percentages of K562 and K562 PR cells in the G1 phase compared with the controls, suggesting that cell-cycle arrest had occurred in the CML cell lines, including in the K562 PR cells. We also found the percentages of sub-G1 phase cells increased within 48 h (Fig. 4D). We then evaluated the gene expressions of *GGCX*, *VKORC1*, and *UBIAD1*. The expressions of *GGCX* and *VKORC1* were increased by co-treatment with asciminib and VK2; however, *UBIAD1* expression was unchanged (Supplemental Fig. 1E).

### Effect of the asciminib and VK2 combination on CML cells

**Fig. 5:**
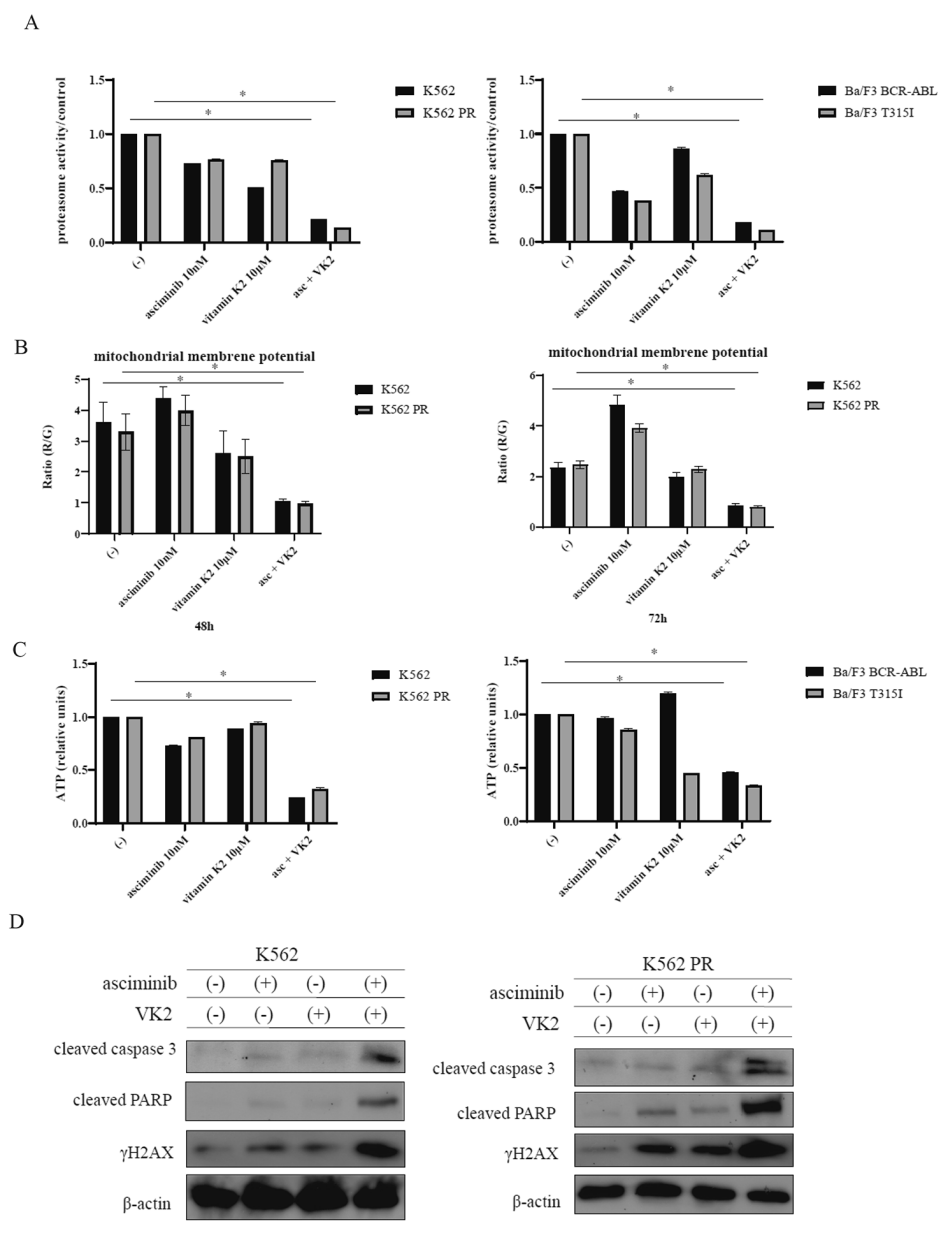
Effects of asciminib and VK2 on CML cell proliferation. **(A)** CML cell lines (K562, K562 PR, Ba/F3 BCR::ABL, and Ba/F3 T315I) were incubated with 10 nM asciminib and/or 10 µM VK2 for 48 h. Proteasome activity was analyzed using the 20 S Proteasome Assay Kit. ^*^p < 0.05 vs. the control. **(B)** CML cell lines (K562, K562 PR) were incubated with 10 nM asciminib and/or 10 µM VK2 for 48 or 72 h. The MMP was analyzed using a Mitochondria Staining Kit. ^*^p < 0.05 vs. the control. **(C)** CML cell lines (K562, K562 PR, Ba/F3 BCR::ABL, and Ba/F3 T315I) were incubated with 10 nM asciminib and/or 10 µM VK2 for 72 h. Intracellular ATP levels were determined using the ‘Cell’ ATP Assay Reagent Ver. 2 Kit. ^*^p < 0.05 vs. the control. **(D)** K562 or K562 PR cells were treated with asciminib and/or VK2 for 24 h. The total extracts were examined by immunoblot analysis with antibodies against γ-H2AX, cleaved caspase 3, cleaved PARP, and β-actin

A previous study showed that c-Abl plays dual roles in the regulation of proteasome homeostasis and activities [21]. According to another study, cells exhibiting BCR::ABL1 activity were associated with higher proteasome levels [22]. Thus, we evaluated 20 S proteasome activity in the CML cell lines using the 20 S Proteasome Assay. Compared with control samples, asciminib and VK2 co-treatment decreased the activity of the 20 S proteasome (Fig. 5A). Monitoring changes in MMP allows for the evaluation of mitochondrial activity, a crucial marker of cellular health [23], and fluorescent dyes have been used to assess MMP to evaluate mitochondrial viability and function. Thus, we evaluated MMP. The red/green fluorescence ratio (R/G) of the dye indicated that the MMP was reduced by co-treatment with asciminib and VK2 in a time-dependent manner (Fig. 5B, Supplemental Fig. 1F). We assessed the amounts of ATP in CML cells since mitochondria are crucial organelles involved in energy production [24]. Intracellular ATP was reduced by co-treatment with asciminib and VK2 (Fig. 5C). Immunoblot analysis revealed that co-treatment with asciminib and VK2 triggered activation of caspase 3, PARP, and γH2AX (Fig. 5D). These results indicated that co-treatment with asciminib and VK2 causes genomic damage.

## Discussion

In the current study, we demonstrated that a combination of new ABL TKIs, asciminib and VK2, significantly enhanced cytotoxicity in CML cells, including those resistant to ponatinib. Over the past 20 years, therapeutic approaches to CML have changed. Since its approval in 2001, imatinib has been successful in producing high remission rates and improving prognoses in the frontline care of CML [3]. However, up to 33% of patients do not achieve optimal responses [25]. The primarily advised monitoring strategy is now measurement of *BCR::ABL1* transcript levels, such as the international scale (IS) of *BCR::ABL1* RNA [26]. In 2013, the ELN incorporated molecular monitoring by including standardized real-time quantitative polymerase chain reaction (qRT-PCR) analysis into their recommendations for the management of CML [27]. An optimal response was *BCR::ABL1* ≤ 10%, ≤ 1%, and ≤ 0.1% at 3, 6, and 12 months after receiving TKI therapy, respectively. More than 10% *BCR::ABL1* at 3 months suggests treatment failure and may imply poor prognoses for these patients [27]. Asciminib can be beneficial for treatment of CML. Although our study showed that the IC50 values of asciminib were similar for K562 and K562 PR cells, caspase 3/7 activity and cytotoxicity differed dramatically between the cell lines. Asciminib may reduce K562 PR cell proliferation when cell cycle arrest occurs; however, induction of apoptosis was not determined at the lower asciminib concentration.

In this work, we found that *GGCX* and *VKORC1* were upregulated, while *UBIAD1* was downregulated, when *BCR::ABL1* > 10% at 3 months after imatinib treatment. GGCX is a vitamin-K-dependent gamma-carboxylase and contributes significantly to the production of vitamin-K-dependent clotting factors [28]. The *VKORC1* gene encodes a key enzyme in the vitamin K cycle and provides instructions for making a vitamin K epoxide reductase enzyme [29]. *UBIAD1* encodes an MK-4 biosynthetic enzyme sometimes referred to as transitional epithelial response protein 1 (TERE1) [30]. The highest expressions of *GGCX* and *VKORC1* have been observed in triple negative breast cancer cell lines and in advanced stages of disease, such as invasive breast cancers, when compared with normal mammary glands [31]. A previous report also demonstrated that the UBIAD1 protein is a tumor suppressor based upon its reduced expression in urological cancer specimens [32]. Therefore, these VK2-related genes may be involved ABL TKI resistance in EMR failure patients. However. it was not clear the gene expressions of *VKORC1* and *GGCX* were increased after asciminib and VK2 treatment. How asciminib and VK2 affect gene expression will be clarified in future research. Our findings suggest a correlation between VK2-related genes and imatinib resistance, and these genes may represent attractive therapeutic targets for CML treatment, particularly for CML patients who have developed imatinib resistance.

We therefore attempted to assess VK2’s anticancer potential in both wild-type BCR::ABL and ponatinib-resistant cells in the current investigation. In this study, VK2 activity was examined at concentrations from 0 to 50 µM, and 10 µM of VK2 was used for combination treatment with asciminib. Although 10 µM of VK2 was high, its blood concentration was increased up to 10 µM by intravenous administration in the clinic. Apoptosis or programmed cell death is a key process regulating cancer development and progression [33]. Our findings showed that asciminib and VK2 enhanced cytotoxicity by activating caspases.

Vitamin K exists in two natural forms: vitamin K1, or phylloquinone, found largely in green leafy vegetables, and VK2, of which meat and cheese are the primary dietary sources [8]. Vitamin K is a necessary nutrient currently being researched for its potential anticancer properties. The combination of VK2 with other chemotherapeutic drugs has been demonstrated to be safe and cost-effective and may be an efficient way to overcome drug resistance. Published reports have shown that vitamin K use in combination therapy improves the efficacy of clinical drugs by promoting apoptosis and cell cycle arrest and overcoming drug resistance [34]. Greater advantages can be obtained if one of the combination agents is a micronutrient advantageous to health. Our research showed that asciminib and VK2 co-treatment resulted in G1 arrest, ABL TKI resistance, and enhanced cytotoxicity. There have been several reports of combinations of asciminib and other compounds. For instance, Eide et al. [35] reported using asciminib and ponatinib together as a therapeutic approach for BCR::ABL1 mutants that are resistant to ABL TKIs. Our modeling implies that combinations of asciminib and VK2 allow for lower asciminib dosages, which may result in a decreased risk of developing ABL TKI resistance and reduce the side effects of asciminib.

## Conclusions

Our findings show that VK2 has a substantial anti-leukemic effect on CML cell lines. The combination of asciminib and VK2 may lead to increased treatment efficacy, multidirectional cellular activity, or suppression of ABL TKI resistance, including that of ponatinib; the combination treatment was effective, even with reduced doses of asciminib (e.g., 10 nM). Treatment of CML, including ABL TKI resistance, using a regimen combining the current standard of care, asciminib, and VK2 appears to be a viable method, although more preclinical and clinical testing is needed.

### Electronic supplementary material

Below is the link to the electronic supplementary material.

Supplementary Material 1

Supplementary Material 2

## Data Availability

The datasets generated and/or analyzed during the current study are available in the NCBI Gene Expression Omnibus (http://www.ncbi.nlm.nih.gov/geo/) repository with the accession numbers GSE130404.

## List of abbreviations

ABL: Abelson
TKI: tyrosine kinase inhibitor
CML: chronic myeloid leukemia
VK2: Vitamin K2
STAMP: specifically targeting the ABL myristoyl pocket
Ph: Philadelphia
CP: chronic phase
DMSO: dimethyl sulfoxide
RPMI1640: Roswell Park Memorial Institute 1640 medium
FCS: fetal calf serum
LDH: lactate dehydrogenase
ELISA: enzyme-linked immunosorbent assay
ATP: adenosine triphosphate
PARP: poly (ADP-ribose) polymerase
EMR: early molecular response
GEO: Gene Expression Omnibus
GGCX: gamma-glutamyl carboxylase
VKORC1: vitamin K epoxide reductase complex subunit 1
UBIAD1: UbiA prenyltransferase domain containing 1
MMP: mitochondrial membrane potential
IS: international scale
qRT-PCR: quantitative reverse transcription-polymerase chain reaction
TERE1: transitional epithelial response protein 1
