# Supplementary figures and images for: Effect of asciminib and vitamin K2 on Abelson tyrosine-kinase-inhibitor-resistant chronic myelogenous leukemia cells
Source: BMC Cancer. 2023 Sep 5;23:827. doi: 10.1186/s12885-023-11304-4 (PMC10478393; doi:10.1186/s12885-023-11304-4)

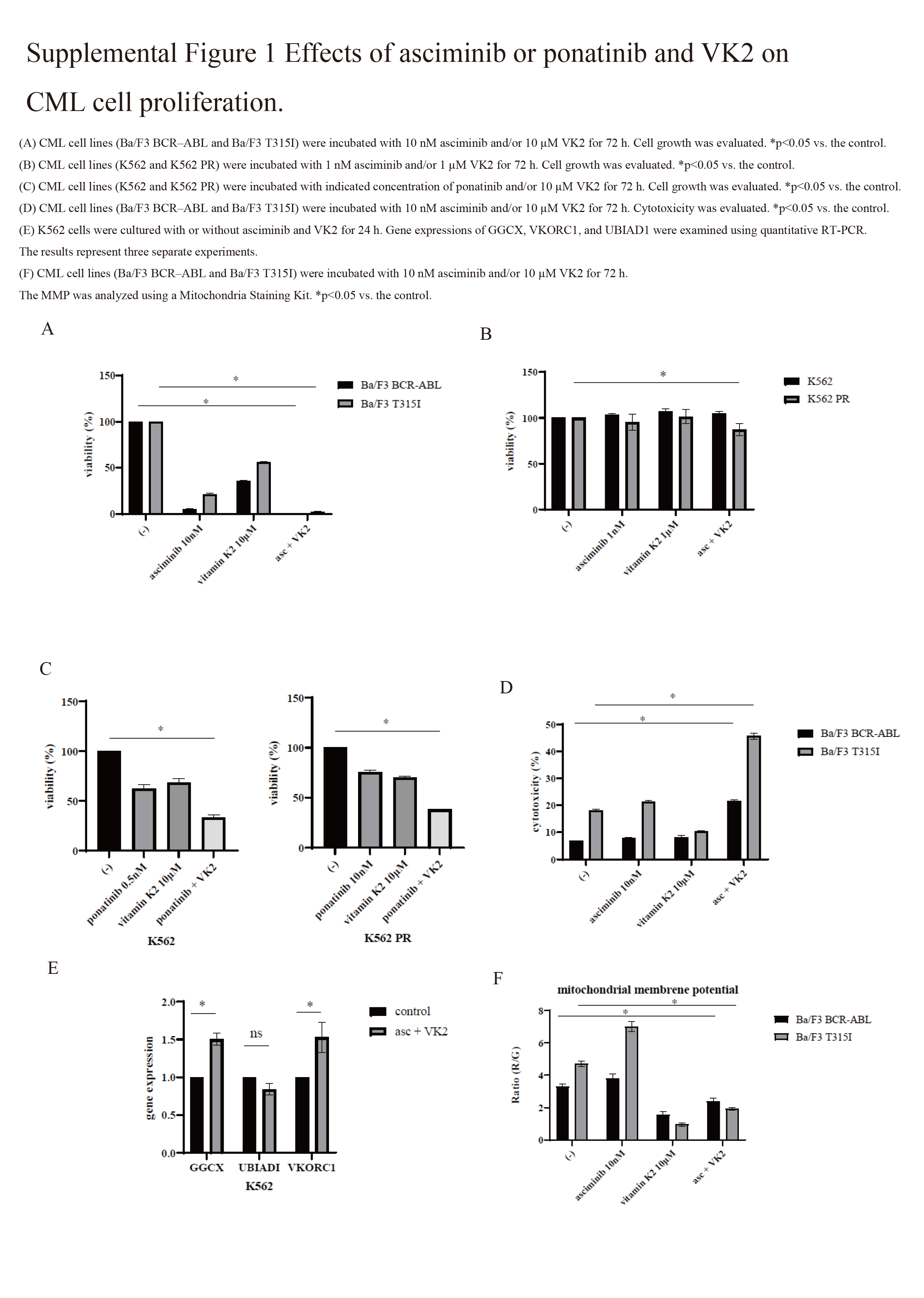

Supplement: Supplementary file 1 — Supplementary Material 1 [file 12885_2023_11304_MOESM1_ESM.tif]

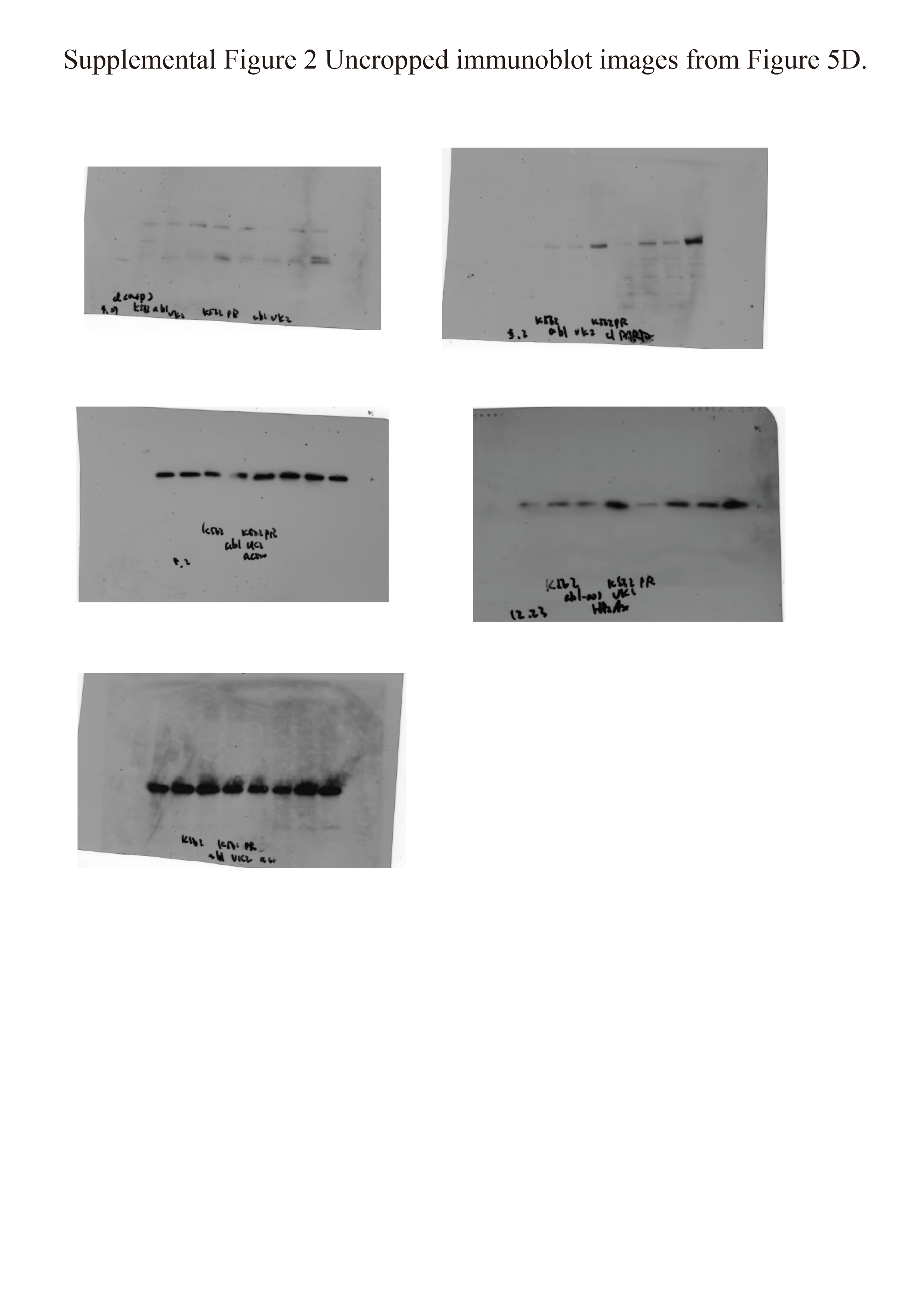

Supplement: Supplementary file 2 — Supplementary Material 2 [file 12885_2023_11304_MOESM2_ESM.tif]
